# An improved deep learning approach for detection of thyroid papillary cancer in ultrasound images

**DOI:** 10.1038/s41598-018-25005-7

**Published:** 2018-04-26

**Authors:** Hailiang Li, Jian Weng, Yujian Shi, Wanrong Gu, Yijun Mao, Yonghua Wang, Weiwei Liu, Jiajie Zhang

**Affiliations:** 1 School of Electronics and Information Technology, Sun Yat-sen University, Guangzhou, 510006 China; 2 College of Information Science and Technology/College of Cyber Security, Jinan University, Guangzhou, 510632 China; 3 TopGene Tech Co., Ltd, Guangzhou, 510627 China; 4 College of Mathematics and Informatics, South China Agricultural University, Guangzhou, 510642 China; 5 School of Automation, Guangdong University of Technology, Guangzhou, 510006 China; 6 Sun Yat-sen University Cancer Center, Guangzhou, 510080 China

## Abstract

Unlike daily routine images, ultrasound images are usually monochrome and low-resolution. In ultrasound images, the cancer regions are usually blurred, vague margin and irregular in shape. Moreover, the features of cancer region are very similar to normal or benign tissues. Therefore, training ultrasound images with original Convolutional Neural Network (CNN) directly is not satisfactory. In our study, inspired by state-of-the-art object detection network Faster R-CNN, we develop a detector which is more suitable for thyroid papillary carcinoma detection in ultrasound images. In order to improve the accuracy of the detection, we add a spatial constrained layer to CNN so that the detector can extract the features of surrounding region in which the cancer regions are residing. In addition, by concatenating the shallow and deep layers of the CNN, the detector can detect blurrier or smaller cancer regions. The experiments demonstrate that the potential of this new methodology can reduce the workload for pathologists and increase the objectivity of diagnoses. We find that 93:5% of papillary thyroid carcinoma regions could be detected automatically while 81:5% of benign and normal tissue could be excluded without the use of any additional immunohistochemical markers or human intervention.

## Introduction

**Figure 1:**
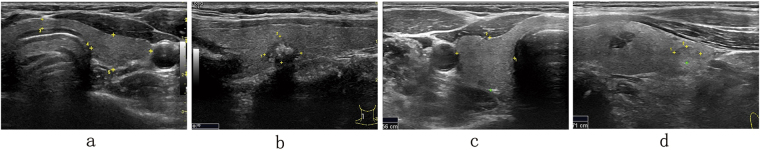
Some ultrasound images of thyroid papillary carcinoma. The cancer regions are are marked by yellow crosses. We can see that cancer regions are blur, vague margin or irregular shape.

Papillary thyroid carcinoma is most common in thyroid carcinoma, accounting for 85%^1^. The diagnosis of papillary thyroid carcinoma is a fundamental step in the process of treatment. Commonly, ultrasound images are monochrome and low-resolution. As shown in Fig. 1, in ultrasound images, cancer regions are usually blurred, vague margin and irregular in shape. Moreover, the features of cancer regions are very similar to normal or benign tissues. As a result, it is difficult to distinguish the cancer region from the analogous tissues. The accuracy of thyroid ultrasound diagnosis is closely depended on the experience and cognitive ability of diagnosticians. Because of the influence of subjective factors, there are usual many differences in judgments of ultrasound images for different diagnosticians. Therefore, the precise ultrasound diagnosis of papillary thyroid carcinoma is a challenging task.

Simulating the human visual mechanism, computer vision is with the advantages of high detection speed and low cost. Computer vision technology is often used in the area of rapid intelligent image processing, such as image classification, object detection and object retrieve^2–4^. In early stage of the computer vision, researchers had focused on designing feature representations for content-based image retrieval (CBIR)^5^ tens of years. The scope included global features^6^ (color, shape, and texture), local features (SIFT^7^ and SURF features^8^) and bag of visual words representations (BOW)^9^. Then, machine learning techniques such as support vector machines (SVM)^10^, k-nearest neighbor (KNN)^11^ and linear discriminant analysis (LDA)^12^ were widely used in image classification. Y Toki, T Tanaka^13^ used the SIFT method to extract the image to identify prostate cancer. With incomplete gland features, comparing to previous methods, the accuracy was improved to 6.3–13.3%. For the color and texture features of biopsy specimens, Niwas, S. I., Palanisamy^14^ used least squares support vector machine (LS-SVM) for the diagnosis of breast cancer. Basavanhally *et al*.^15^ presented a new multiple field of view classifier, with different size of multiple field of view to identify the important features of one image. This method was used for classification of breast cancer pathological images. However, due to the computational costs, the discriminating power of these methods is challenging for identifying definitive features, subset characterization and optimization. In addition, these methods rely on limited manual annotations and are only applicable to fixed feature matching. Once the characteristics (such as twist, flip, illumination, corruption, and so on) changed, the effects of these algorithms will become worse. Therefore, their universality is not strong.

Recently, a promising machine learning approach has made rapid progress in the automatic classification and interpretation of medical image data. During the last few years, Convolutional Neural Network (CNN)^3,16^ becomes one of the most rapidly developing fields in deep learning. As a kind of artificial neural network, it is becoming a research focus in the area of speech analysis^17^ and image recognition^2–4^. The shared weights network structure makes it more akin to human neural networks. Due to shared weights, CNN can reduce the complexity of the network model and reduce the number of weights. CNN has a more significant advantage when the input is a multidimensional image. The whole image is used as the input avoids complex traditional recognition algorithms such as feature extraction and data reconstruction process. The multi-layer perception of CNN is particularly applicable to identify two-dimensional images. It is highly invariant for translation, scaling, skewing and forms of deformation^3^. Lutjanus *et al*.^18^ used CNN to idiomatically identify the features of Sentinel and breast cancer metastasis in the MR image. This method can reduce the workload of the pathologist and increase the objectivity of the diagnosis. The concluded that deep learning holds great promise to enhance the efficacy of prostate cancer diagnosis and breast cancer staging. Angel Cruz-Roaa, Ajay Basavanhally *et al*.^19^ realized automatic segmentation of invasive breast cancer MR images and generated cancer distribution maps by CNN. The authors compared the performance of CNN, hand-crafted image feature extraction method and random forest method. The experiment showed that CNN worked best. Petersen, Kersten and Chernoff *et al*.^20^ proposed a combination of supervised learning and unsupervised learning approach to segment breast density separation and evaluate risk assessment of breast. First, they utilized the deep unsupervised CNN to extract feature of images. Then they used classified images to adjust network weights and offset parameters. This strategy called fine-tuning^3^. The model could be easily extended to many areas of image segmentation and classification. Su *et al*.^21^ used stacked de-noising auto-encoders to detect and segment cell in lung cancer and brain tumors.

In this paper, we analyze the shortcomings of the state-of-the-art object detection network Faster R-CNN for detecting ultrasound image in detail (See Section 0.7). Different from routine images, the cancer regions in ultrasound images are usually blur, vague margin or irregular shape. Facing these problem, we validate the strategies such as layer concatenation and spatial constrained layer. Experimental results show that each strategy can improve the functioning of the detection. Combining all of the strategies yields the best results. In the following, we name this approach CS Faster R-CNN for short.

## Methods

This section presents the mechanism of the CS Faster R-CNN and the pipeline deployed to evaluate the benefits of representation in the task of detection. Before we actually start, we will explain the related concepts such as CNN and Faster R-CNN.

### CNN

**Figure 2:**
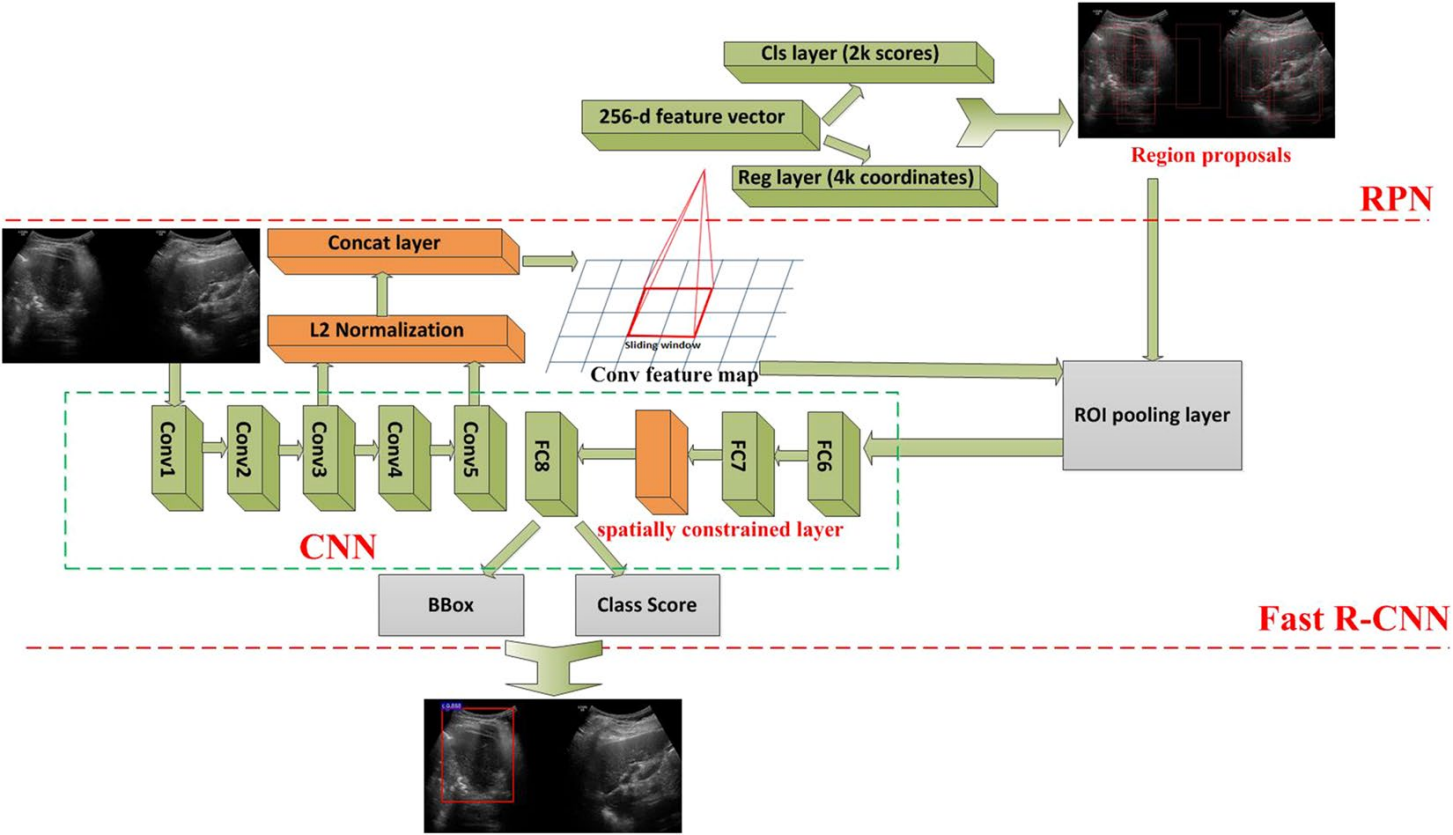
The architecture of proposed CS Faster R-CNN for ultrasound image detection. The simplified CNN model is surrounded by green boxes.

**Figure 3:**
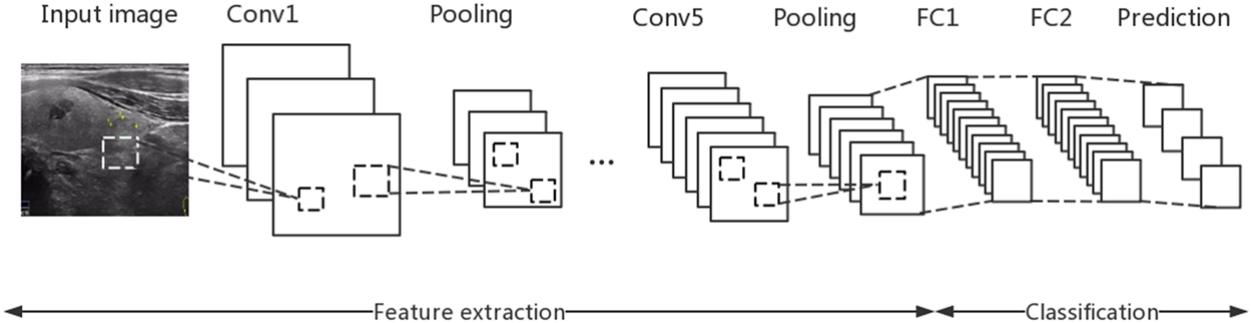
Architecture of ZF model. An 3 channels image with 224*224 is as the input. It is convolved with 96 7*7 filters with a stride of 2 in x and y. Then the process is: (1) processed by rectified linear function (Omit here), (2) using stride 2, max pooled with 3*3 regions, (3) processed by contrast normalized, yielding 96 55*55 feature maps. The following layers 2, 3, 4, 5 perform the same operation, (4) layer 6 and layer 7 are fully connected. They extract features from layer 5 which is as input in the form of vector. The output layer is a softmax function and the “1000” in the figure is the number of classes.

Given *N* training samples ${\{({x}_{i},{y}_{i})\}}_{i=1}^{N}$, where *x* represents annotated region, *y* represents label. Through the training, CNN can estimate a model *F* mapping the relationship between input vectors *x* and output vectors *y*. In detail, The training process includes two phases, the forward propagation phase and the back propagation phase. During the forward propagation phase, when a training sample (*x*_*i*_, *y*_*i*_) is given as input to the network, *x*_*i*_ is transferred from the input layer to the output layer step by step. Finally, we get the output *o*_*i*_. This process can be formulated as,

1 $${o}_{i}={F}_{L}(\mathrm{...}){F}_{2}({F}_{1}({x}_{i}{w}_{1}){w}_{2})\mathrm{...}){w}_{L})$$

where *L* is the number of layers, *w*_*j*_ is the weight vector of the *j*th layer *F*_*j*_. Commonly, we define *F*_*j*_ as a series layers which perform operations such as convolution with kernel function, max pooling or non-linear activation. After a series operations, estimating the weight vectors *w*_1_, *w*_2_, ..., *w*_*L*_ can be solved with the following optimization problem,

2 $$\mathop{{\rm{argmin}}}\limits_{{w}_{1},{w}_{2},\mathrm{...},{w}_{L}}\frac{1}{N}\sum _{i=1}^{N}\ell ({o}_{i},{y}_{i})$$

where $\ell$ is usually defined as cross-entropy cost function. Using back-propagation and stochastic gradient descent methods, we can solve the numerical optimization problem (2). In Fig. 2, the CNN model ZF is framed with the green dashed rectangle and its detailed architecture is shown in Fig. 3. We can see that ZF has 5 convolution layers and 3 full connected layers. Conv5 layer is the top convolution layer, of which is a 3*3 kernel function with a stride of 1. It outputs 256 feature maps with the size of 13*13 which is given as an input for RPN to generate object proposals.

### Faster R-CNN

As the development of R-CNN^22^, Fast R-CNN^23^, Faster R-CNN achieves state-of-the-art performance on pattern analysis, statistical modeling and computational learning visual object classes (PASCAL VOC) datasets^24^. However, both R-CNN and Fast R-CNN need the extra step such as selective search (SS)^25^, Edge boxes^26^ to generate object proposals. Due to just running on CPU, with SS or Edge boxes, extracting all proposals from an image with CPU requires approximately 2 s. In the view of end-to-end, the time-consuming is an obvious bottleneck for R-CNN and Fast R-CNN. By means of powerful feature extraction capability of a neural network (NN), Faster R-CNN integrates Region Proposal Network (RPN) into Fast R-CNN to extract proposals. RPN is a fully Convolutional Networks (FCN), of which the function is to generate high quality region proposals, and each has an confidence score. It simultaneously predicts object bounds and object scores at each position. To generate region proposals, a small network slides over the convolutional feature map output by the top convolutional Layer. Comparing to the extra step SS or Edge boxes, RPN can share full-image convolutional features with the Fast R-CNN, enabling nearly cost-free region proposals. It simultaneously predicts object bounds and confidence scores at each position. In Faster R-CNN, both Fast R-CNN and RPN are trained together using a simple alternating optimization. For the very deep VGG-16 model^27^, Faster R-CNN has a frame rate of 5fps on a GPU, while achieving state-of-the-art object detection accuracy on PASCAL VOC 2007 (73.2% mAP) and 2012 (70.4% mAP).

### Improved Faster R-CNN for ultrasonic image detection

In view of problems referred in 0.7, we investigate a series strategies to make Faster R-CNN suitable to detect ultrasound images. As shown in Fig. 2, in CNN used in Faster R-CNN, the conv3 layer and conv5 layer of ZF is concatenated and normalized. In addition, we add a spatial constrained layer before the output layer. These strategies will be introduced with detail in the following. In our research, we mainly aim at improving the CNN used in Faster R-CNN.

#### Layer concatenation

In CNN, with the deeper layers, the reception fields become bigger. Therefore, deeper layer has smaller-scale values while shallower layer has bigger-scale values. Due to the large scale difference, it is difficult for the following layers to adjust and tune the weights. If we directly concatenate the tensors of the conv3 layer and the conv5 layer of ZF model, the “big” feature in the conv5 layer will override the “small” feature in the conv3 layer. As a result, the output of the results is likely to express “big” feature and ignore a “small” feature.

According to^28^, these two tensors need the normalized operation. In the process of Faster R-CNN training, the system can automatically learn the scaling factor of each layer. Therefore, the normalization operation can keep the stability and precision of the system^28^. As shown in Fig. 2, we apply L2 normalization to tensors in the conv3 layer and the conv5 layer. We make the normalization within each pixel in the pooled feature map tensor. After the normalization, scaling is applied on each tensor independently as:

3 $$\hat{X}=\frac{X}{{\Vert X\Vert }_{2}}$$

4 $${\Vert X\Vert }_{2}={(\sum _{i=1}^{d}|x{|}^{2})}^{\frac{1}{2}}$$

where *X* is the original pixel vector, $\hat{X}$ is the normalized pixel vector and *d* stands for the number of channels in each RoI pooling tensor.

The scaling factor ${\Upsilon }_{i}$ is then applied to each channel for every ROI pooling tensor:

5 $${y}_{i}={\Upsilon }_{i}{\hat{x}}_{i}$$

During training, the update for the scaling factor ${\Upsilon }_{i}$ and input *X* is calculated with back-propagation and chain rule:

6 $$\frac{\partial l}{\partial \hat{X}}=\frac{\partial l}{\partial Y}\cdot \Upsilon $$

7 $$\frac{\partial l}{\partial X}=\frac{\partial l}{\partial \hat{X}}(\frac{I}{{\Vert X\Vert }_{2}}-\frac{X{X}^{T}}{{{\Vert X\Vert }_{2}}^{3}})$$

8 $$\frac{\partial l}{\partial {\Upsilon }_{i}}=\sum _{{y}_{i}}\frac{\partial l}{\partial {y}_{i}}{\hat{x}}_{i}$$

where *Y* = [*y*_1_, *y*_2_, ..., *y*_*d*_]^*T*^.

#### Spatial constrained layer

During the training phase, CNN only extracts features from the annotated regions. However, because of the pathologists’ experience and cognitive level, the annotated regions are often subjective or even inaccurate. In addition, the cancer regions depend on their residing regions which are hard to define. That is to say, the output *y* may not only depend on the input *x* alone, but also on the topological domain region on which it is residing. In order to extract features from the unknown residing regions, as shown in Fig. 2, we add a Spatial constrained layer before the output layer. We define Ω as the residing region which the output *y* depends on. The Spatial constrained regression model *m* can be expressed as,

9 $$y=m({\rm{\Omega }};\theta (x))$$

where *θ* (*x*) is an unknown parameter vector which can be estimated. Here we suppose that *m* is known a priori. As in Fig. 2, we can estimate *θ* (*x*) by

10 $$\theta (x)={F}_{L-1}({x}_{L-2};{w}_{L-1})$$

where *x*_*L*−2_ is an output of the (*L* − 2) th layer of the network. When a image including a annotated region with height *H*, width *W* is given as an input into network, *y* ∈ [0, 1]^*H*′×*W*′^ can be denoted as a probability map between *y* ∈ [0, 1] and the spatial domain Ω = [1, ..., *H*′]×[1, ..., *W*′], *H*′ > *H*, *W*′ > *W*. The *i* th element of *y*_*i*_, *i* = 1, ..., |Ω| is defined as,

11 $${y}_{i}=\{\begin{array}{ll}\frac{1}{1+({\Vert {c}_{i}-{c}_{o}\Vert }_{2}^{2})/2} & {\Vert {c}_{i}-{c}_{o}\Vert }_{2} > d,\\ 0 & others,\end{array}$$

where *c*_*i*_ represents the coordinates of *y*_*i*_, *c*_*o*_ represents the the center of the cancer region within Ω. We define *d* as constant radius of cancer region which can be estimated with experiments.

As shown in Fig. 2, the predicted output $\hat{y}$ is the output of the Spatial constrained layer. Through training, we can get the probability map (11). Following (11), the *i* th element of the predicted output ${\hat{y}}_{i}$ can be expressed as

12 $$\begin{array}{l}{\hat{y}}_{i}=m({c}_{i};{\hat{c}}_{o})=\{\begin{array}{ll}\frac{1}{1+({\Vert {c}_{i}-{c}_{o}\Vert }_{2}^{2})/2} & {\Vert {c}_{i}-{c}_{o}\Vert }_{2} > d,\\ 0 & others,\end{array}\end{array}$$

where ${\hat{c}}_{o}\in {\rm{\Omega }}$ is an estimated center of the probability mask. In our experiments, we set *d* in (11) and (12) to 150 pixels. ${\hat{c}}_{o}=(r;q)$ can be estimated in the (*L* − 1) th layer using (10). *r*, *q* can be defined as

13 $$\begin{array}{l}r=(H^{\prime} -1)\cdot sigm({w}_{L-1,r}\cdot {x}_{L-2})+{b}_{r}+1,\\ q=(W^{\prime} -1)\cdot sigm({w}_{L-1,q}\cdot {x}_{L-2})+{b}_{q}+1,\end{array}$$

where *w*_*L*-1_, _*r*_,*w*_*L*-1,*q*_ denote the weight vectors and *b*_*r*_, *b*_*q*_ denote the bias variables, and *sigm*(⋅) denotes the sigmoid function. To learn all the variables (i.e., weight vectors and bias values) in the network, we solve (2) using the following cross-entropy loss function:

14 $$\ell (y,\hat{y})=-\,\sum _{j}[{y}_{i}\,\mathrm{log}({\hat{y}}_{i})-(1-{y}_{i})(1-\,\mathrm{log}({\hat{y}}_{i}))]$$

## Experiments

### Collecting Data

We collect the ultrasound images of 300 cases from the Department of head and neck of Sun Yat-sen University Cancer Center. All experimental protocols were approved by the Ethics Committee of the Sun Yat-sen University Cancer Center, and were conducted in accordance with the Good Clinical Practice guideline. Informed consent was obtained from each patient for their consent to have their information used in research without affecting their treatment option or violating their privacy. These ultrasound images are taken among 2012–2014, from 53 males and 247 females at the age of 10–85 years. 250 cases were diagnosed with papillary thyroid cancer and underwent surgery. The other 50 cases were diagnosed with thyroid normal. In order to ensure the accurate of the data, all cases have complete diagnostic records, ultrasound reports and treatment schemes. In addition, all the training samples are images mentioned in ultrasound reports which contain annotations and their description of representations. That is to say, these images have ready-made annotations. Each case has 5–25 ultrasound images and the sum is 4670. The each ultrasound image of the diagnosed case has 1–3 cancer regions. We select the ultrasound images of 200 diagnosed cases as training samples. The remaining 100 cases (50 diagnosed cases and 50 normal cases) are used for test samples. This 50 diagnosed cases include 1027 ultrasound images. Keeping the original aspect ratio, we set all the width of training samples to 1000 px.

**Figure 4:**
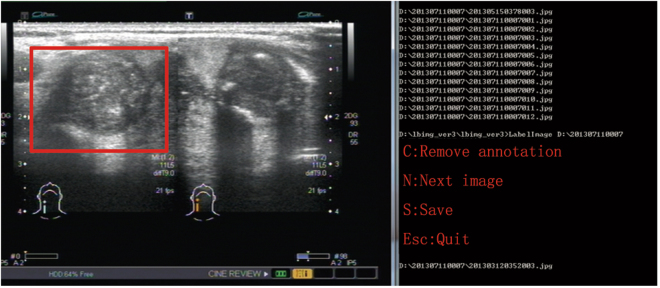
The screenshot of the software interface in the annotation process.

To ensure objectivity, we only provide the annotator with original ultrasound images without any annotations. With the help of a tagging software, two experienced physicians annotate the training samples with the red rectangular box. Testing samples with cancer regions are also annotated to generate the ground truths. The screenshot of the software interface in the annotation process is shown in Fig. 4. The rectangular box will be dropped if the shortest side is less than 2 mm. In addition, the rectangle box must completely surround the cancer region and as small as possible. As referred in Section 0.3, all the images used for annotation are selected from ultrasound reports containing ready-made annotation and their description of representations generated by the previous physicians. In addition, following the corresponding ultrasound reports, another two physicians reviews the annotated images. With the help of the original annotations in ultrasonic reports, the experienced physicians’ annotation and the inspectors’ verification, we can ensure the accuracy of the annotation. In this way, we annotate 6727, 1881 regions in training samples and testing samples respectively. Finally, we make XML files for all labeled training samples according to the requirement of Faster R-CNN.

### Annotation

#### Training

Our experiments are done on Ubutun 14.04 64 bit installed with Python version of Faster R-CNN (The Matlab and Python version of Faster R-CNN can be downloaded at: https://github.com/rbgirshick/), using 32 G Nvidia TITAN X (Pascal) acceleration. Because of the lack of training samples, we fine-tune Faster R-CNN using an image dataset VOC2007 database. Specially, we utilize CNN model ZF which is pre-trained with the VOC2007 database. Theoretically, fine-tuning all convolutional layers will result in the best performance. To balance time consumption and efficiency, we fine-tune the weights of all the layers, except the first two convolutional layers. During fine-tuning, we take the approximate joint training scheme in^29^ to train the RPN and detector simultaneously using multitask loss.

As referred in Section 0.2.1, we modify the source code of Faster R-CNN to normalize the conv3 and conv5 layers and concatenate the features pooled from them. As illustrated in^28^, the scale used after the features being concatenated could be either refined or fixed. Here we use a fixed scale of 4700 for the entire blob, both in the training and test phases.

In the training phase, we set iteration numbers [50000, 25000, 50000, 25000], using a fixed learning rate of 0.0001. With the above parameters and data, training a CS Faster R-CNN model tkkes about 12 hours. Then we use the model to test training samples. The output regions whose confidence scores are above 0.8 while their IoU values with any ground-truth annotation are less than 0.3, are considered as the hard negatives. A selected region proposal would be regarded as a cancer if the confidence score is higher than 0.8. Results showed that CS Faster R-CNN is a real-time system, detecting an image takes an average of 0.15 s.

## Results

In the following, true positive, false positive, true negative, false negative, true positive rate, false positive rate, true negative rate and false negative rate, are called TP, FP, TN, FN, TPR, FPR, TNR and FNR respectively for short. To further gain deep insights of the improvements obtained by our proposed method, we conduct more additional experiments for ablation studies as listed in Table 1, where we aim to examine the effectiveness and contributions of different strategies used in the proposed method. The 10-fold cross validation is used to estimate the performance of all the strategies. We present results for splits on per image (i.e., the training set and the validation set do not share the same image). Table 2 presents the results that pool each of the ten folds together. In Table 2, using ID3, that is CS Faster R-CNN, 93.5% of papillary thyroid carcinoma regions can be detected automatically while 81.5% of benign and normal tissue can be excluded without using any additional immunohistochemical markers or human intervention. Therefore, the potential of this new methodology could reduce the workload for pathologists and increase the objectivity of diagnoses.

**Table 1:** Combination of strategies one by one.

	Layer concatenation	Spatial constrained layer
ID1	No	No
ID2	Yes	No
ID3	Yes	Yes

**Table 2:** Performance of strategies.

	TP	TPR	FP	FPR	TN	TNR	FN	FNR
Ground truth	1881	1	—	—	359	1	—	—
ID1	1633	0.868	104	0.289	255	0.711	248	0.132
ID2	1694	0.900	79	0.218	280	0.782	187	0.100
ID3	1759	**0.935**	67	**0.185**	292	**0.815**	122	**0.065**

To better validate the effectiveness of each strategy, we compare the performance of strategies one by one. As shown in Table 1, ID1 does not use any strategy. ID2 uses the strategy of layer concatenation. ID3 uses the strategy of layer concatenation and spatial constrained layer. Following we will discuss the performance of each strategy in detail.

### Results of the ablation experiments

#### Layer concatenation

**Figure 5:**
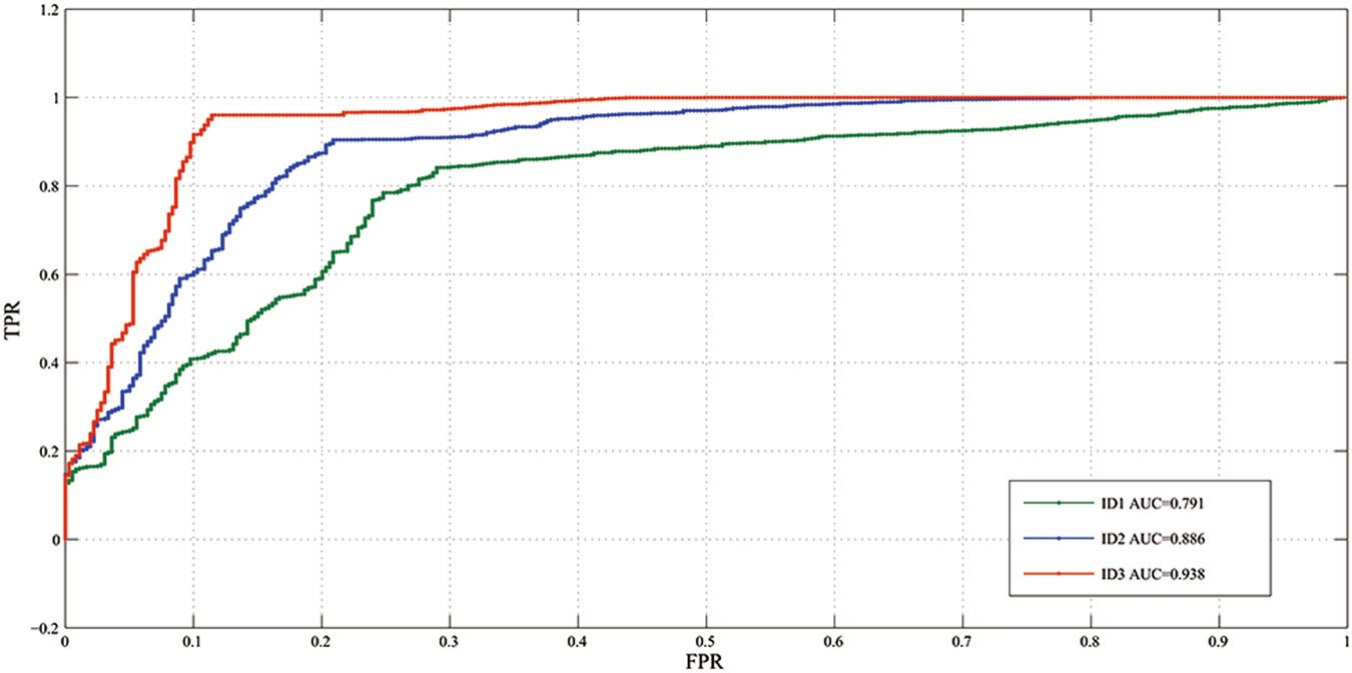
The comparison of the ROCs and areas under the curves (AUCs).

**Figure 6:**
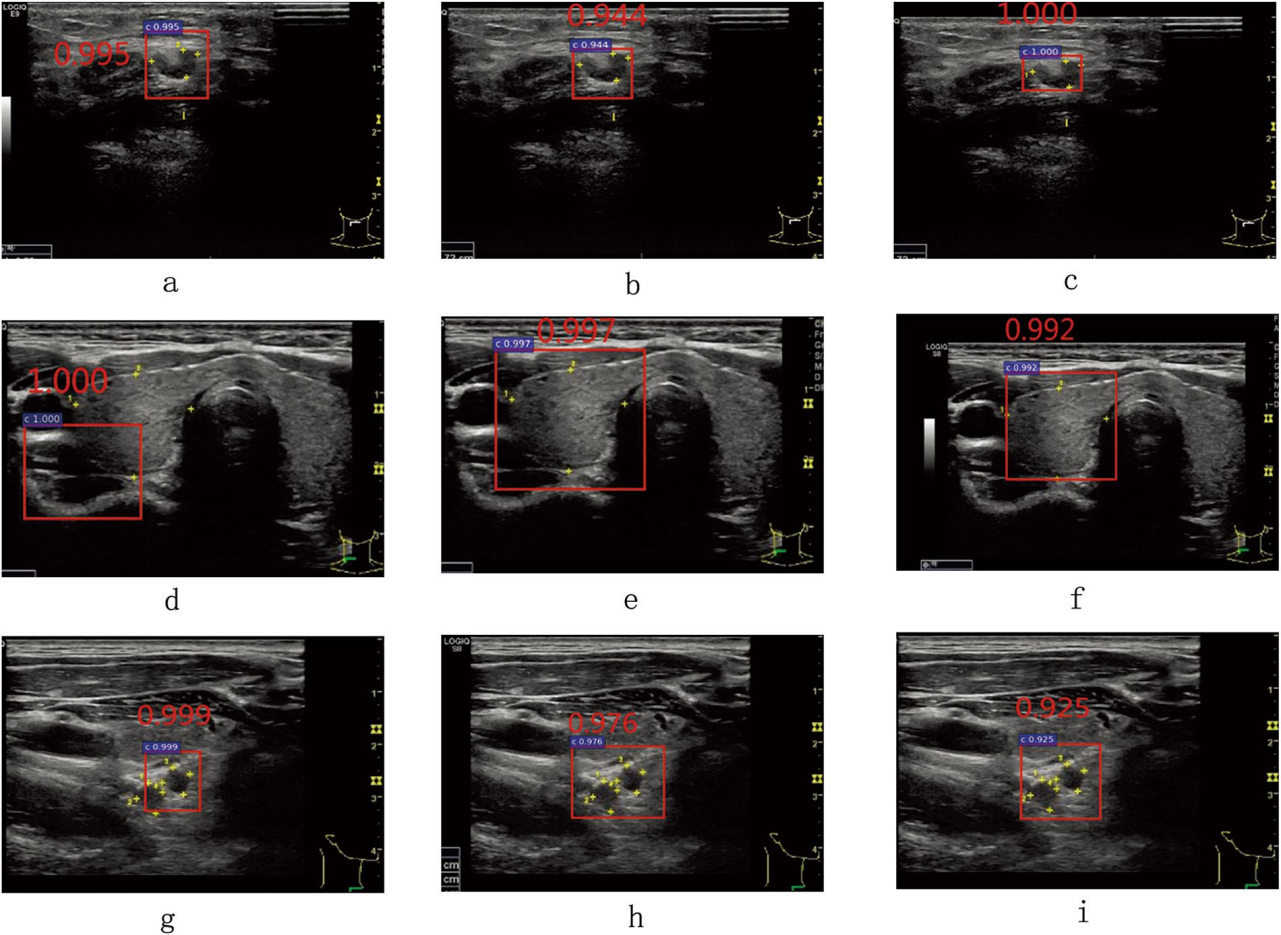
Some correct detection results. The same row is the detection results of the same image. The first, second and third column are the detection results of ID1, ID2 and ID3 respectively. The ground truth of cancer regions are marked with yellow stars. The detection results are framed by the red rectangular boxes.

**Figure 7:**
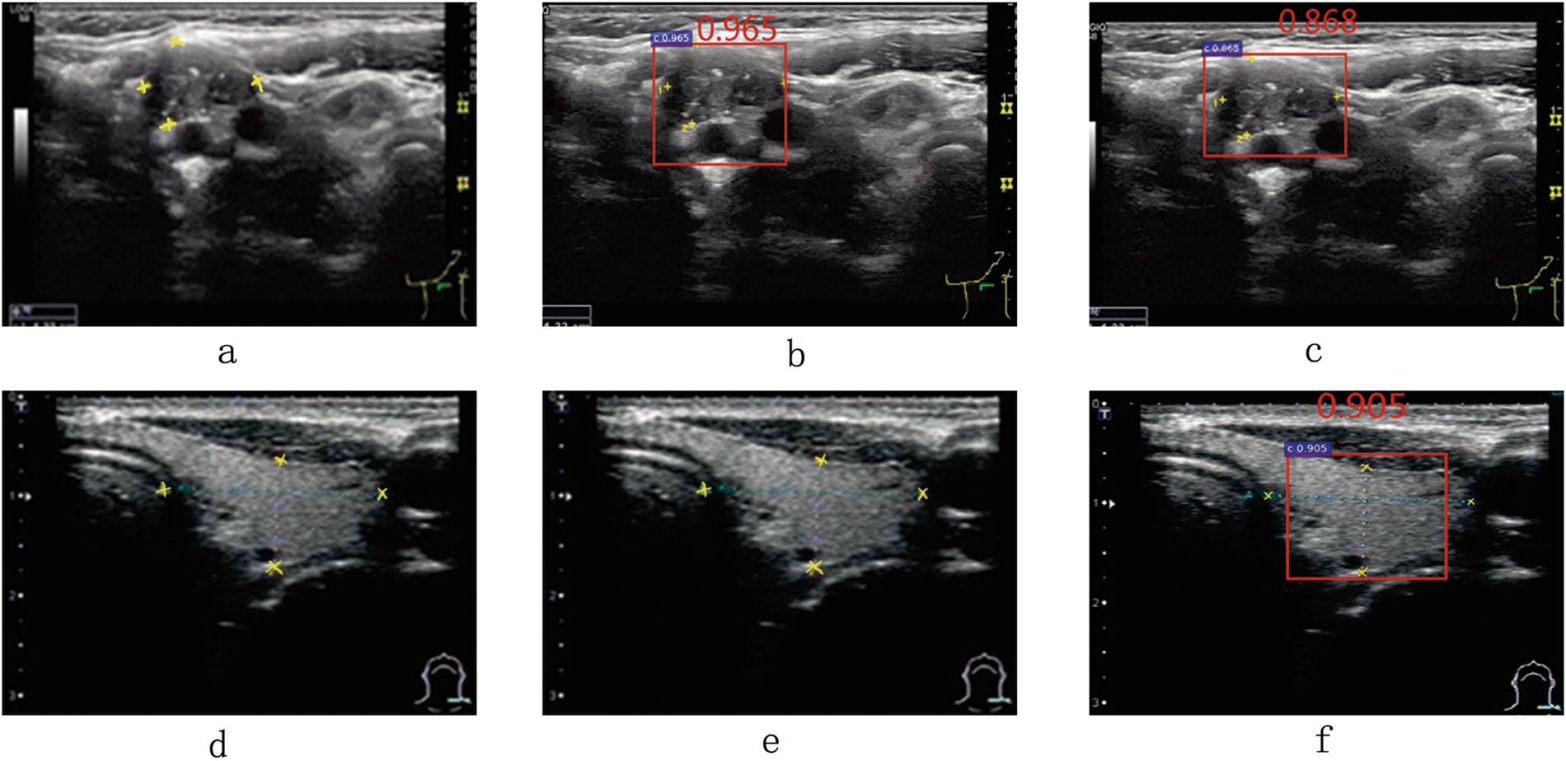
Some FN (**a**,**d**,**e**) and TP (**b**,**c**,**f**) detection results. The same row is the detection results of the same image. The first, second and third column are the detection results of ID1, ID2 and ID3 respectively. The ground truth of cancer regions are marked with yellow stars. The detection results are framed by the red rectangular boxes.

**Figure 8:**
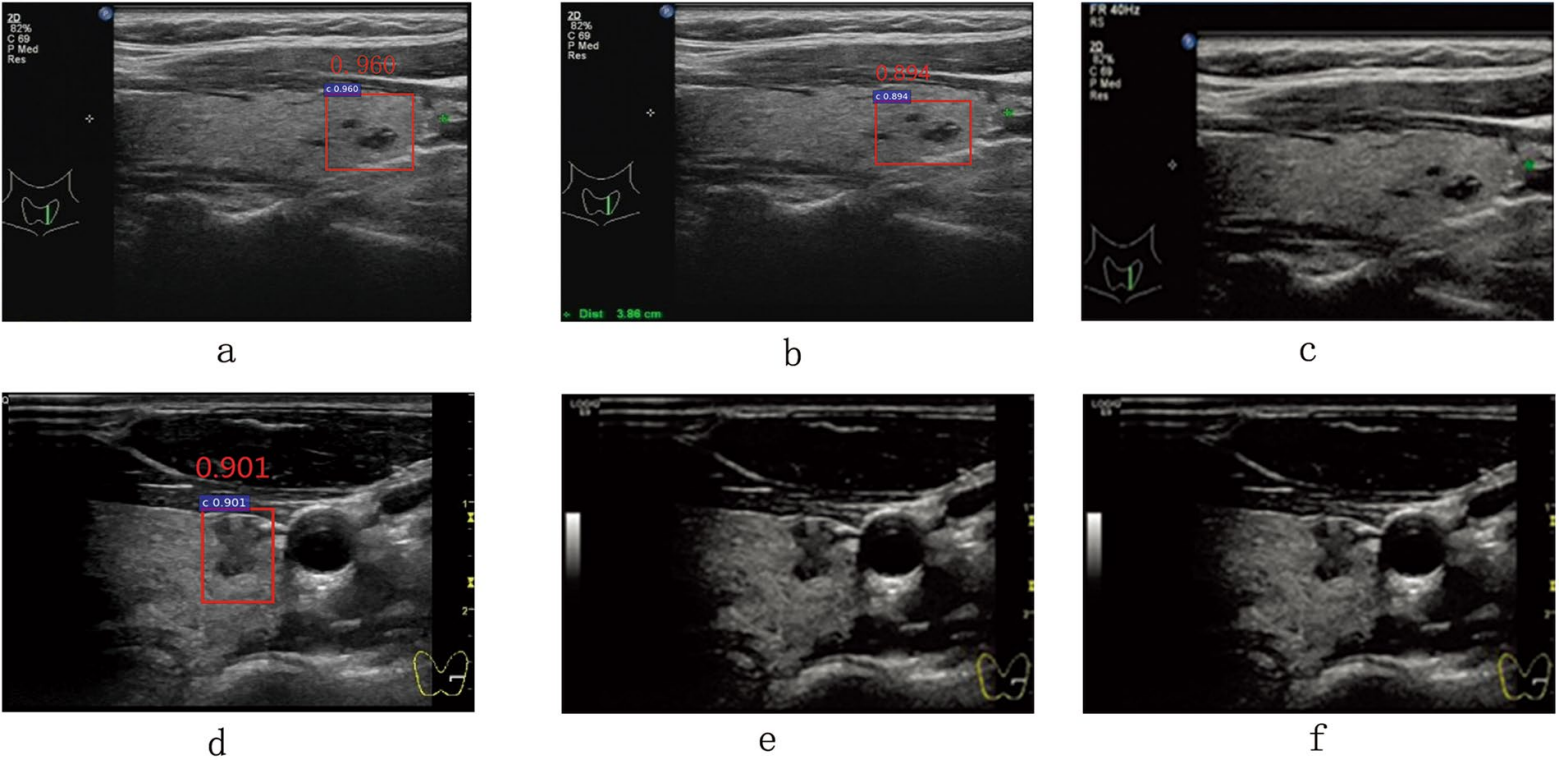
Some FP (**a**,**b**,**d**) and TN (**c**,**e**,**f**) detection results. The same row is the detection results of the same image. The first, second and third column are the detection results of ID1, ID2 and ID3 respectively. The ground truth of cancer regions are marked with yellow stars. The detection results are framed by the red rectangular boxes.

As in Fig. 5, the receiver operating characteristic curve (ROC) of ID2 using spatial constrained layer is closer to the top left corner than ID1. From Table 2, comparing to ID1, the TPR and TNR of ID2 increase by 3.2%, 7.1% respectively. Intuitively, as shown in first two columns of Fig. 6, for the same ultrasound image, both ID1 and ID2 can identify the correct cancer regions. However, comparing to ID1, the detection result of ID2 is more close to ground truth. Fig. 7a and Fig. 7b are detection results of ID1 and ID2 respectively, we find that ID1 can not identify the cancer regions while ID2 can. However, both ID1 and ID2 can not identify the cancer regions in Fig. 7d and Fig. 7e. It shows that ID2 still needs to be further improved. In addition, we find that both Fig. 8a and Fig. 8b generate FP in the same image. However, the classification score of ID2 is smaller that ID1. In Fig. 8d and Fig. 8e, we find that ID2 can eliminate FP while ID1 can not. In summary, comparing to ID1, ID2 can eliminate more FN and FP results effectively. These results confirm the statements mentioned in the first paragraph of Section 0.7. Due to the RoI pooling mechanism, the original Faster R-CNN cannot capture more local texture of cancer regions, especially for the monochromatic and low-resolution ultrasound images. Using the strategy of layer concatenation, our approach can extract both local and whole texture features of the cancer regions and get a better performance.

#### Spatial constrained layer

As in Fig. 5, the ROC of ID3 using layer concatenation and spatial constrained layer is obviously closer to the top left corner than ID1 and ID2. From Table 2, comparing to ID2, the TPR and TNR of ID2 increase by 3.50%, 3.30% respectively. Intuitively, as shown in Fig. 7b and Fig. 7c, comparing to ID2, ID3 gets a more accurate rectangular results which is closer to ground truth. In Fig. 7d–Fig. 7f, only ID3 can identify the cancer region correctly. In Fig. 8a–Fig. 8c, both ID1 and ID2 generate the FP results while ID3 can get the TN result correctly. As referred in Section 0.2.2, by using the strategy of spatial constrained layer, our approach can fully utilize the features of the residing environment around the annotated cancer regions during training. Therefore, ID3 yields the better performance than ID2.

### Results of comparison with approaches based on SVM

For image classification, SVM has the best performance among machine learning approaches^10^. In our study, we compare CS Faster R-CNN with some state-of-the-art ultrasound image classifier base on SVM. For an unbiased comparison, we only compare their classification performance rather than regional detection performance. Both CS Faster R-CNN and existing methods are trained and validated with the same samples. For SVM approaches, it is worth noting that the training samples are no longer annotated the cancer regions, but just are annotated by classification label, e.g., 1 represents positive sample, −1 represents negative sample. For test results of CS Faster R-CNN, we decide it is tested positive if at least one positive region is contained, or it is negative. Note that all experiments were carried out using 10 fold cross-validation, i.e., one tenth of the cases were used for testing and the rest for training.

We compare our results with several approaches based on SVM such as Moradi, M *et al*.^30^, Virmani *et al*.^31^, Acharya *et al*.^32^, Acharya *et al*.^33^, Tsiaparas *et al*.^34^ and Güler *et al*.^35^. Table 3 shows the results of the comparison. We used the publicly available Matlab implementation of the SVM algorithms named LIBSVM^36^ as the basic platform because the source codes of these studies are not public. We used the optimal implementation as proposed by the authors respectively. As shown in Table 3, for both TPR and TNR, CS Faster R-CNN has the best performance. Acharya *et al*.^33^ has the highest TPR and TNR among the approaches based on SVM. Comparing to Acharya *et al*.^33^ CS Faster R-CNN has increases of 2.7%, 4.7% to TPR and TNR respectively. Obviously, CS Faster R-CNN can identify more correct samples, especially the negative samples. For SVM, it is difficult to judge whether a region is positive or negative because the features of cancer region are very similar to normal or benign tissues in ultrasound images. However, through a series of strategies, CS Faster R-CNN can get better performance than state-of-the-art approach based on SVM.

**Table 3:** Performance comparison with state-of-the-art approaches based on SVM.

	TPR	FPR	TNR	FNR
CS Faster R-CNN	**0.935**	**0.185**	**0.815**	**0.065**
Moradi M *et al*.^30^	0.889	0.306	0.694	0.111
Virmani *et al*.^31^	0.901	0.285	0.715	0.099
charya *et al*.^32^	0.866	0.329	0.671	0.134
Acharya *et al*.^33^	0.904	0.234	0.766	0.086
Tsiaparas *et al*.^34^	0.847	0.361	0.639	0.153
Güler *et al*.^35^	0.799	0.378	0.622	0.201

## Discussion

Many studies have begun to use the state-of-the-art object detection network Faster R-CNN for image classification and detection. However, it is rarely used in ultrasonic image detection. Unlike daily life photos, ultrasound images have some shortcomings which result that using CNN directly for detection of ultrasound images is not feasible. First, we known that deep learning needs a large amount of labeled training data. Ultrasound images are limited and difficult to obtain. Second, as shown in Fig. 1, ultrasound images are usually blur, vague margin or irregular shape. In particular, it is a considerable challenge to distinguish malignant tumor tissue and benign tumor tissue. Finally, cancer tissues lodge in the surrounding environment, and it is difficult for us to identify their boundaries. For the first question, as referred in^37^, though there are substantial differences between natural and medical images which may advise against knowledge transfer, fine-tuning a CNN that has been pre-trained with a large set of labeled natural images still outperforms or, in the worst case, performs as well as a CNN trained from scratch. Therefore, we fine-tune the CNN used in Faster R-CNN with public image dataset VOC2007. For the second question, in order to detect the detail of the cancer regions, we must thoroughly identify their local texture features. However, Faster R-CNN can not extract local texture features well due to the following reasons. The Regions of Interesting (RoI) pooling layer of Faster R-CNN only uses feature maps of the deepest convolution layer. As reported in^38^, as the layer becomes deeper, the reception fields become larger. Therefore, deeper layers have larger-scale values while shallower layers have smaller-scale values. For instance, given that the overall stride of the conv5 layer in the ZF model is 16, once the object size is less than 16 pixels, Faster R-CNN can no longer project the RoI pooling region proposal. From the viewpoint of the feature visualization, as the author pointed out in^39^, conv5 layer (the deepest layer) captures entire features of object. Therefore, Faster R-CNN cannot capture more local texture of object due to the RoI pooling mechanism, and it is difficult for the Faster R-CNN to extract local texture features from low-resolution images. Inspired from^39^, we concatenate conv3 layer and conv5 layer to enable the RoI to pool both local and global features. For the last question, in order to extract features from the unknown residing regions, we add a spatial constrained layer before the output layer.

**Figure 9:**
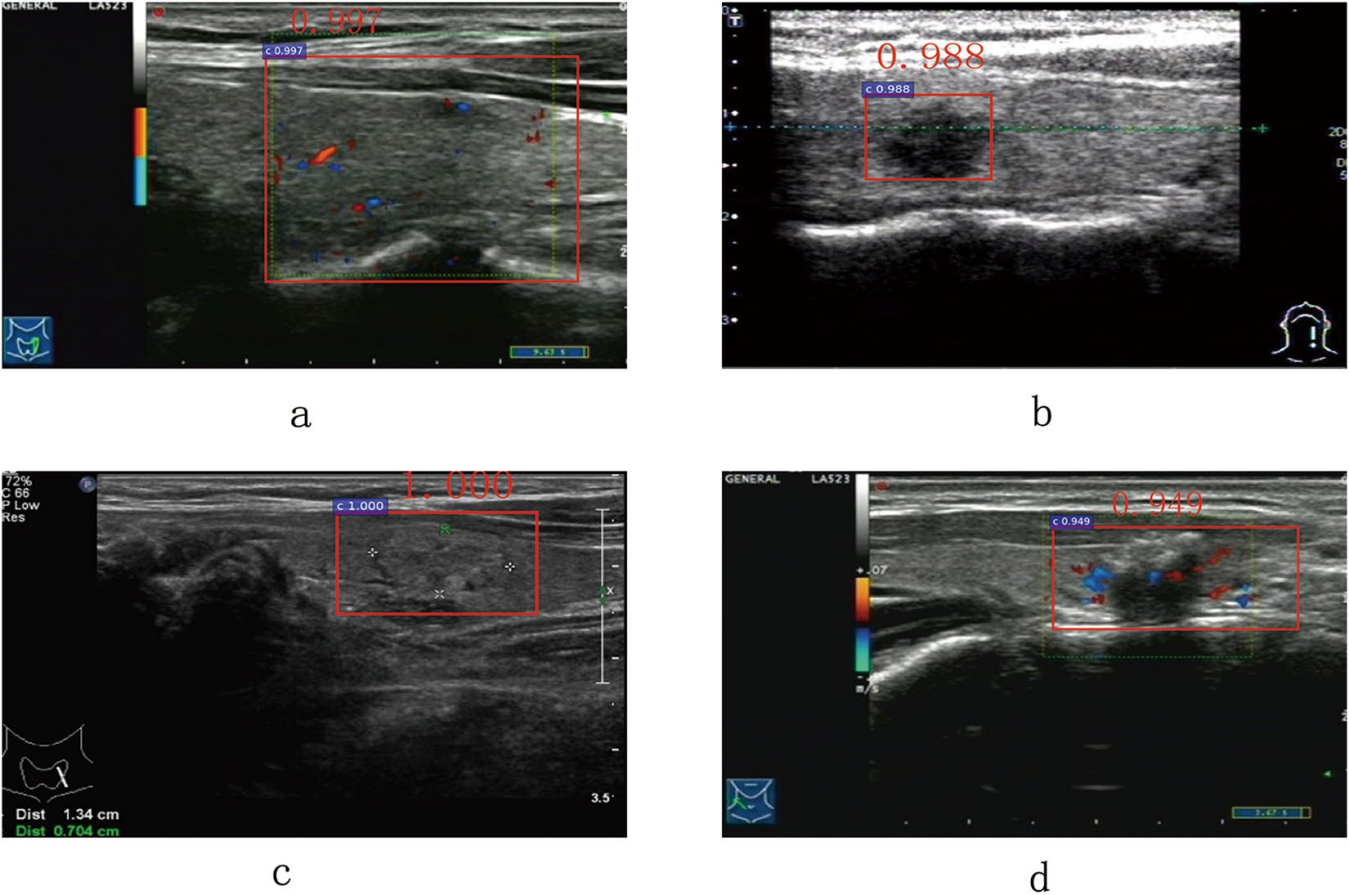
Some detection result of our approach, we can find that CS Faster R-CNN can identify images which contains blur (**a**), low-resolution (**b**), vague margin (**c**) and irregular shape (**d**) caner regions.

By using layer concatenation, we concatenated the features pooled from conv3 and conv5 layer of ZF^39^ used in Faster R-CNN. This strategy could enhance the ability of detector to capture more detail features of the RoI, especially for low-resolution images. Experiments showed that this strategy could increase the TPR by 3.3%. By using spatial constrained layer, the detection could extract the features of surrounding host environment in which the cancer regions are residing, increasing the TPR and the TNR by 6.3%, 7.5% respectively. As seen in Fig. 5 and Table 2, combining the strategies of layer concatenation and spatial constrained layer, ID3, that is CS Faster R-CNN, can dramatically improve the detection performance, exceeding any single strategy. Fig. 9 presents that ID3 has good recognition results for blur, low-resolution, vague margin and irregular shape caner regions. In terms of efficiency, as shown in Table 4, using the same training sample referred in Section 0.3, ID1, ID2 and ID3 take about 8.5 hours, 11 hours and 12 hours respectively. This is because ID2 takes extra about 2.5 hours to compute the layer connection and normalization. In the same way, ID3 takes extra about 1 hour to compute the spatial constrained layer. For testing a ultrasound image, ID1, ID2 and ID3 take about 0.10 s, 0.13 s and 0.15 s respectively. That is, all of them are real-time detection system.

**Table 4:** Performance of efficiencies.

	Training time (hour)	Detecting time (s)
ID1	8.5	0.10
ID2	11	0.13
ID3	12	0.15

Experiments show that we have achieved good results by adopting these strategies. We will further investigate why not concatenate conv4 and conv5 layer or other two layers. As mentioned in^39^, The conv1 layer just responds to some blocks or lines which have no semantic features. The conv2 Layer responds to some corners and other edge/color conjunctions which still have no semantic features. The conv3 Layer has more complex invariances, capturing similar textures (e.g. mesh patterns) which have semantic features. The conv4 Layer shows significant variation, and is more class-specific: dog faces or bird legs. The conv5 Layer shows entire objects with significant pose variation, e.g. keyboards or dogs. Therefore, the conv1 and conv2 layers can not represent the local texture features. Under the same experimental conditions, we have compared the performances of the using single layers and the using different layer concatenations. The results are as shown in Table 5. In Table 5, we can see that in all the individual layers, the conv5 layer has the best effect, the TPR reaches 0.897, 0.238 (23.8%) higher than the conv4 layer alone with the second-best TPR 0.659. In all the layer concatenations, we can see that the concatenation of the conv3 and conv5 layers has the best effect, the TPR reaches 0.935, 0.025 (2.5%) higher than the concatenation of the conv4 and conv5 layers with the second-best TPR 0.910. However, in all the layer concatenations, if the conv5 layer is not involved, the best effect is generated by the concatenation of the conv3 and conv4 layers, the TPR is only 0.691. Therefore, we can conclude that the conv5 layer works best when we use an individual layer. If we use the layer concatenations, the concatenation of the conv3 and conv5 layers works best. The reason is that the conv5 layer is necessary for extracting the whole texture features of cancer regions. The detector can not identify the cancer regions if there is no whole texture features. The conv3 layer extract the local texture features of the cancer regions, and the concatenation of the whole texture features and the local texture features can achieve the best effect.

**Table 5:** Performances of the using single layers and the using different layer concatenations.

	TPR	FPR	TNR	FNR
1	0.416	0.612	0.388	0.584
2	0.479	0.596	0.404	0.521
3	0.514	0.504	0.496	0.486
4	0.659	0.365	0.635	0.341
5	0.897	0.276	0.724	0.103
3 & 5	**0.935**	**0.185**	**0.815**	**0.065**
4 & 5	0.910	0.205	0.795	0.090
2 & 5	0.901	0.265	0.735	0.099
1 & 5	0.872	0.277	0.723	0.128
3 & 4	0.691	0.329	0.671	0.309
2 & 4	0.641	0.410	0.590	0.359
2 & 3	0.512	0.509	0.491	0.488
1 & 3	0.462	0.558	0.442	0.538
1 & 2	0.407	0.619	0.381	0.593

The numbers in first column represent the convolutional layers. ‘&’ represents concatenation.

## Conclusion

This study investigates the strategies to improve the ability of Faster R-CNN to detect cancer regions in thyroid papillary carcinoma images. Facing fewer training samples and blurry cancer regions, we validate the strategies such as layer concatenation and Spatial constrained layer. Experimental results show that each strategy can improve the functioning of the detection. Combining all of the strategies yields the best results. In future, we will investigate new strategies to detect more kind of cancer region considering the context. In addition, we will further study how to generate a exhaustive and practical diagnostic report.
